# HUST bearing: a practical dataset for ball bearing fault diagnosis

**DOI:** 10.1186/s13104-023-06400-4

**Published:** 2023-07-06

**Authors:** Nguyen Duc Thuan, Hoang Si Hong

**Affiliations:** School of Electrical and Electronic Engineering, Hanoi University of Science and Technology, No. 1 Dai Co Viet Road, Hanoi, Vietnam

**Keywords:** Dataset, Bearing fault, Fault diagnosis

## Abstract

**Objectives:**

The rapid growth of machine learning methods has led to an increase in the demand for data. For bearing fault diagnosis, the data acquisition is time-consuming with complicated processes. Existing datasets are only focused on only one type of bearing, which limits real-world applications. Therefore, the objective of this work is to propose a diverse dataset for ball bearing fault diagnosis based on vibration.

**Data description:**

In this work, we introduce a practical dataset named *HUST bearing*, which provides a large set of vibration data on different ball bearings. This dataset contains 99 raw vibration signals of 6 types of defects (inner crack, outer crack, ball crack, and their 2-combinations) on 5 types of bearing (6204, 6205, 6206, 6207, and 6208) at 3 working conditions (0 W, 200 W, and 400 W). Each vibration signal is sampled at a rate of 51,200 samples per second for 10 s. The data acquisition system is elaborately designed with high reliability.

## Objective

Electric motors are indispensable equipment in manufacturing plants. They often have to work continuously and are subjected to a variety of heavy loads, and operate in harsh environments. Therefore, bearings in electric motors become vulnerable components. Statistics show that electric motor faults related to bearings account for nearly 50% of the total number of common faults [1]. The consequences when a bearing fault occurs are severe, including the destruction of mechanical structures, production stoppage, and costly repair [2]. For these reasons, the early detection and diagnosis of bearing faults is an urgent task and plays a crucial role in electrical machine health monitoring.

When a bearing defect appears, it generates abnormal signals such as vibrations, sound waves, and temperature [3]. In particular, the vibration signal often contains useful information, so it is widely used to detect and diagnose bearing faults [4]. Vibration signal-based methods are usually approached in three approaches, i.e., physical model-based, signal processing, and machine learning-based methods [5]. Machine learning methods have shown superiority over conventional methods when giving results with high accuracy, especially thanks to deep learning models [6]. The rapid growth of machine learning methods has led to an increase in the demand for data. These data are collected from defective bearings to support the learning process.

In practice, the data acquisition process takes time with difficult and complicated processes [7]. Data acquisition tasks include defect generation on bearings, measurement system setup, and data recording. Fortunately, many open-access datasets have been published for scientists and engineers to research and develop. Some of the most popular bearing fault data sets are CWRU [8], Paderborn [9], IMS [10], and Pronostia [11]. However, the common point of the mentioned above data sets is to focus on only one type of bearing and this is the limitation for the in-real applications. This is the motivation for us to propose a novel dataset for ball bearing fault diagnosis with a high diversity compare to previous datasets.

## Data description

### Defects generation

In general, there are two ways to create bearing defects: artificial and natural. However, the natural fault generation process is relatively complex, so the bearing defects in our dataset are artificially generated. Our object is electric motors operating at low loads, so the bearings used to create defects consist of ID 6204, 6205, 6206, 6207, and 6208 ball bearings from KG Bearing India. As for the fault types, from our observation, when a bearing appears with one defect, it easily leads to another defect on another component due to mechanical interaction. Therefore, our data set includes common single faults such as inner race fault, outer race fault, and ball fault. In addition, double faults are also incorporated as inner and outer faults, inner and ball faults, and outer and ball faults (see Data file 1 in Table 1). We use the wire-cutting method to create a 0.2 mm micro-crack width, simulating the initial state of the fault. We only created one early-stage fault size because we believe that early diagnosis of faults is much more important than diagnosing the extent of the fault so that 0.2 mm fault is enough. Descriptions for the bearing dimensions (inner diameter in mm, outer diameter in mm, ball diameter in mm, and ball number) are: (20, 47, 7.6, 8) for 6204, (25, 52, 7.8, 9) for 6205, (30, 62, 9.0, 9) for 6206, (35, 72, 11.0, 9) for 6207, and (40, 80, 12.0, 9) for 6208.

### Test bench setup

The basic layout of the test bench is demonstrated in Data file 2 in Table 1. The test bench consists of a 750 W (1 HP) induction motor driving a multi-step shaft and a powder brake of Leroy Somer. The multi-step shaft means the shaft with multiple step changes in diameter. The motor is controlled by an inverter and the powder brake plays as a simulated load. Furthermore, a torque transducer and a dynamometer are also mounted to the shaft to monitor the load and velocity of the motor. Defective bearings are mounted to different types of housings and these housings can be flexibly replaced on the multi-step shaft. On the bearing, an accelerometer of PCB 325C33 is installed in the vertical direction to measure vibration.

### Data records

The data acquisition system includes an acceleration sensor, a measurement module, a chassis, and Labview software. All listed components built a fully automated measurement system for the data acquisition step. Vibration analog signals from the sensor are sent to the NI-9234 sound and vibration input module which provides an analog-to-digital conversion. Then converted digital signals from NI-9234 are synchronized and conditioned by NI-CompactDAQ chassis before transferring to the software using USB connectivity. In the software, vibration signals are presented, analyzed, and acquired. The configuration of the measurement module and chassis is set in the software.

The description of all 99 data files is shown in Data set 1 in Table 1. It includes vibration data of 27 prototype defective bearings and 3 healthy bearings at three load conditions of 0 W, 200 W, and 400 W. In addition, the introduced dataset provides 30 vibration data of the defective bearings at a run-up time of 5 s. The vibration during run-up may early alert the adverse condition of the bearings, especially for high-power electric motors which consume starting time. Each data file consists of one or two letters representing the bearing condition, followed by the bearing type and loading condition. For example, for the file I402.mat, the letter I indicates **I**nner race fault, the digit 4 indicates bearing type 620**4**, and digit 2 indicates **2**00 W loading condition (see also abbreviations and [12]). In the data files, “data” is the vibration signal, “fs” is the shaft frequency in Hz, “rpm” is the shaft angular velocity in RPM during run-up time, “ru” and “ru_raw” are the vibration signals during run-up time. It is worth noting that all data are collected with a high sample rate of 51.2 kHz in which these data can record signal changes in detail.

**Table 1:** Overview of data files/data sets

Label	Name of data file/data set	File types (file extension)	Data repository and identifier (DOI or accession number)
Data set 1	HUST bearing dataset	MAT files (.mat)	Mendeley Data (10.17632/cbv7jyx4p9) [13]
Data file 1	defects	PNG file (.png)	Mendeley Data (10.17632/cbv7jyx4p9) [13]
Data file 2	testbench	PNG file (.png)	Mendeley Data (10.17632/cbv7jyx4p9) [13]

## Limitations

- The proposed dataset is missing 2 cases which are ball fault (B) and inner and ball fault (IB) for bearing ID 6204.
- The intensity of defect in different bearings is different because the depth of the crack is not the same. Therefore, the mean/peak amplitude of the vibration signal is markedly different in different cases.
- This dataset does not take into account environmental noise as well as mechanical imperfections.

## Data Availability

The data described in this Data note can be freely and openly accessed on the Mendeley Data repository under 10.17632/cbv7jyx4p9. Please see Table 1 and references [13] for details and links to the data.

## Abbreviations

ID: Identification
I: Inner
O: Outer
B: Ball
IO: Inner and outer
IB: Inner and ball
OB: Outer and ball
